# Supplementary material for: Clair3-trio: high-performance Nanopore long-read variant calling in family trios with trio-to-trio deep neural networks
Source: Brief Bioinform. 2022 Jul 17;23(5):bbac301. doi: 10.1093/bib/bbac301 (PMC9487642; doi:10.1093/bib/bbac301)
Supplement: supplementary-bib_bbac301 [file supplementary-bib_bbac301.docx]

**Supplementary Materials**

Clair3-Trio: high-performance Nanopore long-read variant calling in family trios with Trio-to-Trio deep neural networks

Junhao Su, Zhenxian Zheng, Syed Shakeel Ahmed,Tak-Wah Lam, Ruibang Luo

[Supplementary Figures 3](#_Toc106279208)

[Supplementary Figure 1. SNP benchmarking results on the GIAB trio. 3](#_Toc106279209)

[Supplementary Figure 2. INDEL benchmarking results on the GIAB trio. 4](#_Toc106279210)

[Supplementary Figure 3. Phased alignment visualization for *de novo* variants that fail to be detected by Clair3-Trio. 5](#_Toc106279211)

[Supplementary Figure 4. SNP benchmarking results on the GIAB trio when only parents have diverse depth. 6](#_Toc106279212)

[Supplementary Figure 5. INDEL benchmarking results on the GIAB trio when only parents have diverse depth. 7](#_Toc106279213)

[Supplementary Figure 6. Phased alignment visualization of a variant that correctly were called by Clair3-Trio at a low coverage. 8](#_Toc106279214)

[Supplementary Tables 9](#_Toc106279215)

[Supplementary Table 1. HG002 trio multiple-coverage benchmarking results. 9](#_Toc106279216)

[Supplementary Table 2. HG002 trio’s *de novo* variants failed to be detected by Clair3-Trio. 11](#_Toc106279217)

[Supplementary Table 3. HG005 trio benchmarking results. 12](#_Toc106279218)

[Supplementary Table 4. HG002 trio multiple-coverage benchmarking results at Guppy5 data. 12](#_Toc106279219)

[Supplementary Table 5. HG002 trio multiple-coverage benchmarking results while child’s coverage fixed at 60x. 15](#_Toc106279220)

[Supplementary Table 6. HG002 trio benchmarking results while child’s coverage have lower coverage. 17](#_Toc106279221)

[Supplementary Table 7. Runtime and memory usage of different tools. 17](#_Toc106279222)

[Supplementary Notes 18](#_Toc106279223)

[Summary of methods tested that showed no or negligible improvement 18](#_Toc106279224)

[Data sources 18](#_Toc106279225)

[Reference genomes 18](#_Toc106279226)

[GIAB Truth Variants 19](#_Toc106279227)

[Oxford Nanopore (ONT) Sequencing Data 19](#_Toc106279228)

[Commands 20](#_Toc106279229)

[Read alignment using Minimap2 (v2.17-r941) 20](#_Toc106279230)

[BAM subsampling using Samtools (v1.10) 20](#_Toc106279231)

[Coverage calculation using Mosdepth (v0.2.9) 20](#_Toc106279232)

[Clair3-Trio model training 20](#_Toc106279233)

[Running Clair3-Trio (v0.1) 20](#_Toc106279234)

[Running Clair3 (v0.1-r6) 21](#_Toc106279235)

[Running PEPPER (r0.4) 21](#_Toc106279236)

[Benchmarking using hap.py (v0.3.12) 22](#_Toc106279237)

[Merge VCF with BCFtools (v1.12) 22](#_Toc106279238)

[Benchmarking using RTG tools (v3.12.1) 22](#_Toc106279239)

[Computing the TP and FP of number of de novo variants 23](#_Toc106279240)

# Supplementary Figures


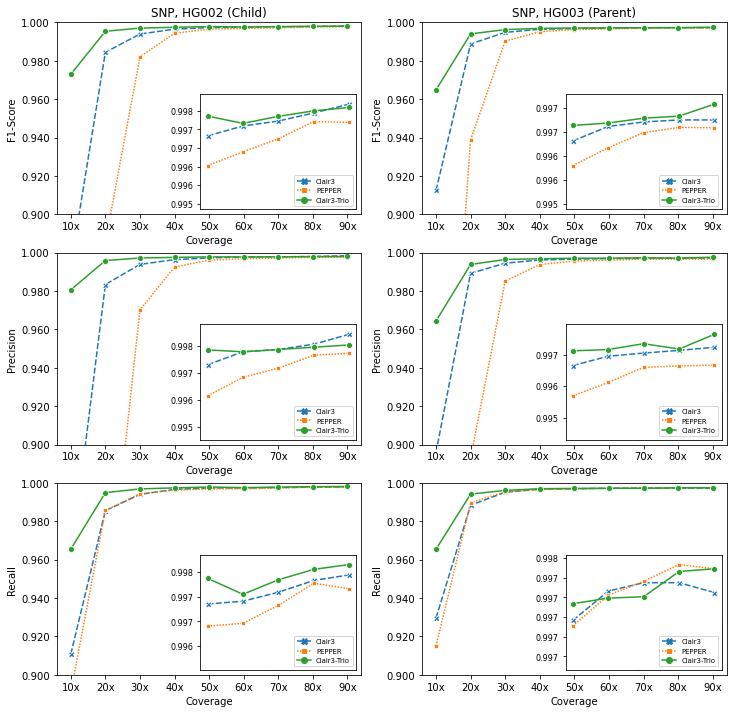


## Supplementary Figure 1. SNP benchmarking results on the GIAB trio.

The SNP’s F1-score, Precision and Recall for HG002 (child, left) and HG003 (parent, right) of different tools at coverages from 10x to 90x.


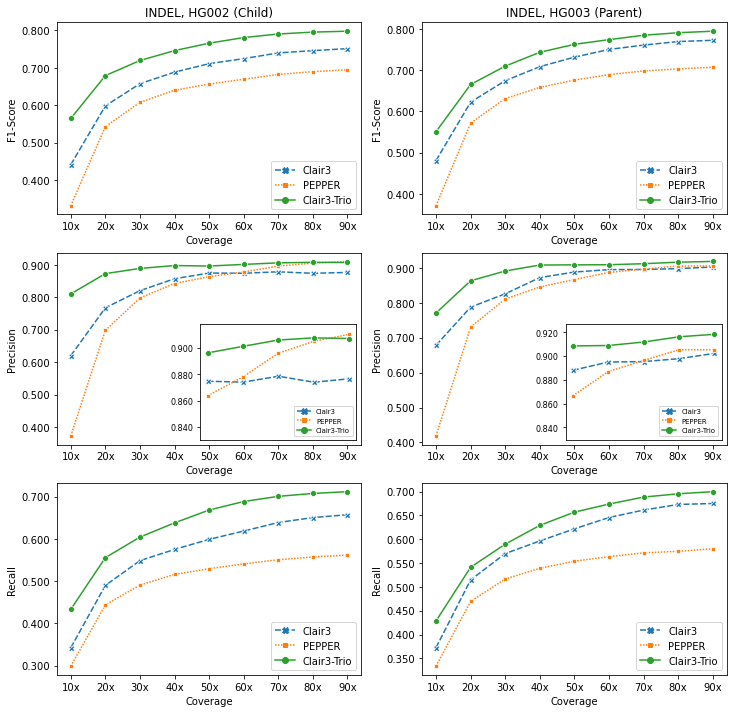


## Supplementary Figure 2. INDEL benchmarking results on the GIAB trio.

The INDEL’s F1-score, Precision and Recall for HG002 (child, left) and HG003 (parent, right) of different tools at coverages from 10x to 90x.

##
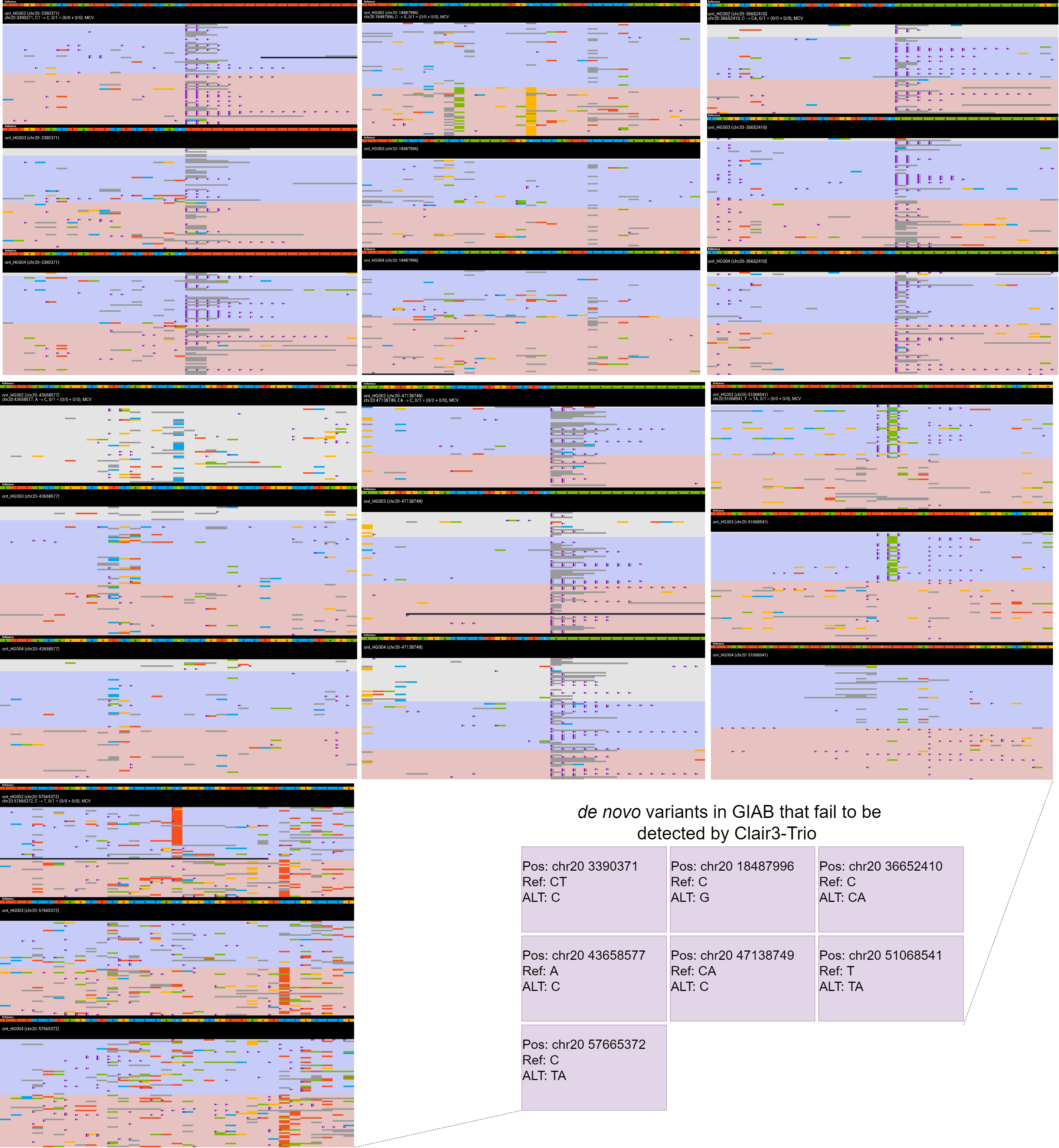
Supplementary Figure 3. Phased alignment visualization for *de novo* variants that fail to be detected by Clair3-Trio.

For each visualization, from the top to bottom in the figure are alignments for HG002 (child), HG003 (parent1), and HG004 (parent2). The A, C, G, T, insertion, and deletion are in green, blue, yellow, red, purple dots, and grey, respectively.


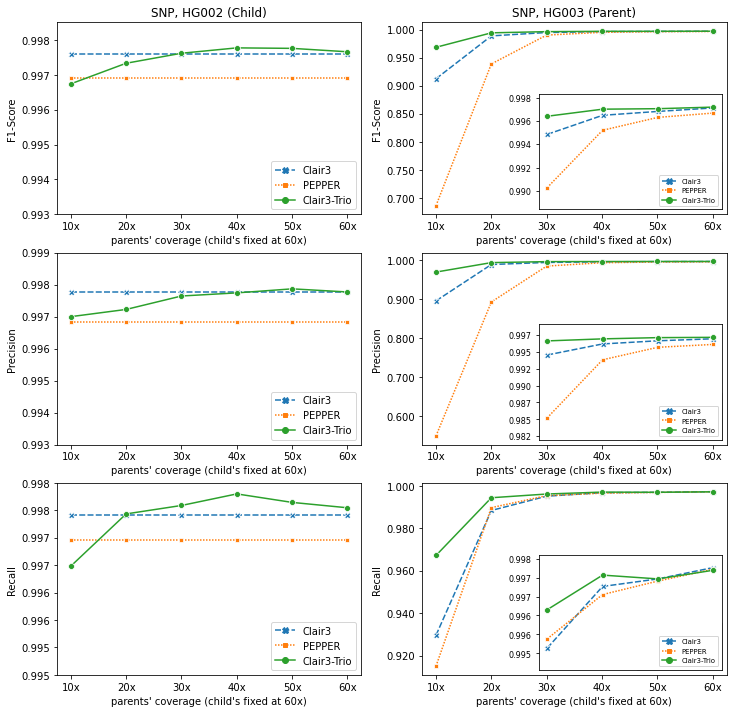


## Supplementary Figure 4. SNP benchmarking results on the GIAB trio when only parents have diverse depth.

The SNP’s F1-score, Precision and Recall for HG003 (parent) of different tools at coverages from 10x to 60x while child’s coverage fixed at 60x is shown.


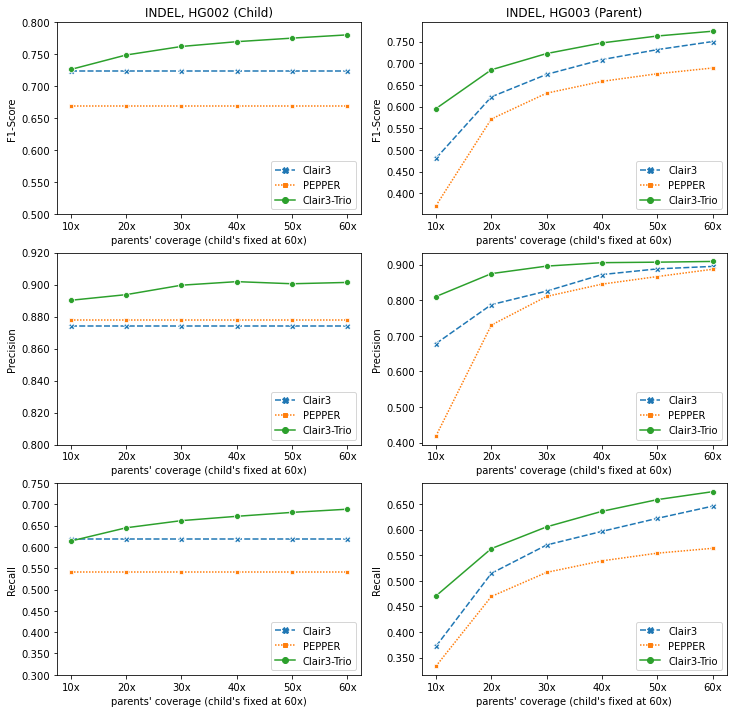


## Supplementary Figure 5. INDEL benchmarking results on the GIAB trio when only parents have diverse depth.

The INDEL’s F1-score, Precision and Recall for HG003 (parent) of different tools at coverages from 10x to 60x while child’s coverage fixed at 60x is shown.


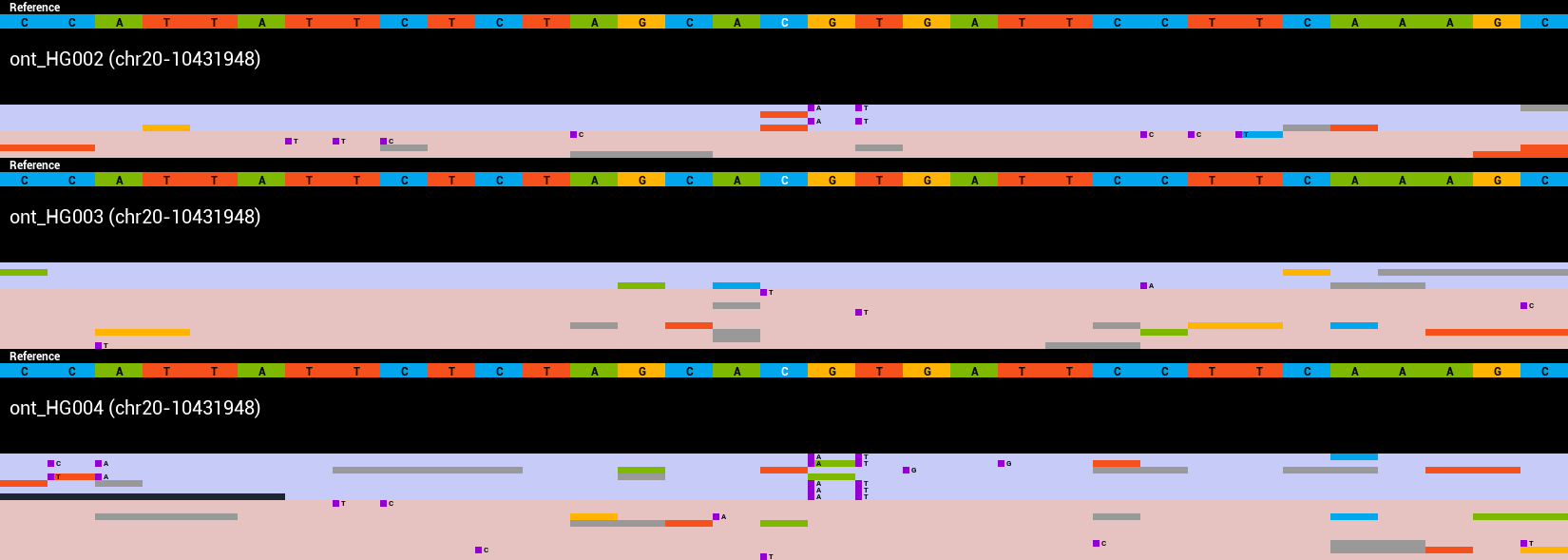


## Supplementary Figure 6. Phased alignment visualization of a variant that correctly were called by Clair3-Trio at a low coverage.

Clair3-Trio is the only tool that correctly called the variants at Chr20:10431948 as HG002: C,CAT (0/1), HG003: C,C (0/0), and HG004 C,CAT (0/1), with only 10x of ONT data. Both Clair3 and PEPPER incorrectly called the variants as HG002: C,T (0/2), HG003: C,C (0/0), and HG004: C,CAT (0/1). From the top to bottom in the figure are alignments for HG002 (child), HG003 (parent1), and HG004 (parent2). The A, C, G, T, insertion, and deletion are in green, blue, yellow, red, purple dots, and grey, respectively.

# Supplementary Tables

## Supplementary Table 1. HG002 trio multiple-coverage benchmarking results.

| Coverage | Tool | Sample | Overall | | | SNP | | | INDEL | | | Insertion | | | Deletion | | | # of MCV | *de novo* TP | *de novo* FP |
| --- | --- | --- | --- | --- | --- | --- | --- | --- | --- | --- | --- | --- | --- | --- | --- | --- | --- | --- | --- | --- |
|  |  |  | Precision | Recall | F1-Score | Precision | Recall | F1-Score | Precision | Recall | F1-Score | Precision | Recall | F1-Score | Precision | Recall | F1-Score |  |  |  |
| 10x | Clair3 | HG002 | 82.24% | 83.33% | 82.78% | 83.89% | 91.08% | 87.33% | 62.00% | 34.25% | 44.12% | 66.85% | 34.89% | 45.85% | 57.95% | 33.65% | 42.58% | 48345 | 33 | 12979 |
|  | Clair3 | HG003 | 87.95% | 85.63% | 86.77% | 89.59% | 92.96% | 91.24% | 67.83% | 37.24% | 48.08% | 70.41% | 39.19% | 50.35% | 65.34% | 35.42% | 45.94% |  |  |  |
|  | Clair3 | HG004 | 86.98% | 84.91% | 85.93% | 88.70% | 92.40% | 90.51% | 66.02% | 36.14% | 46.71% | 66.12% | 37.47% | 47.83% | 65.92% | 34.92% | 45.66% |  |  |  |
| 20x | Clair3 | HG002 | 96.34% | 91.80% | 94.02% | 98.34% | 98.55% | 98.45% | 76.76% | 49.01% | 59.82% | 77.85% | 49.76% | 60.71% | 75.73% | 48.31% | 58.99% | 28411 | 35 | 1787 |
|  | Clair3 | HG003 | 97.07% | 92.61% | 94.79% | 98.92% | 98.84% | 98.88% | 78.76% | 51.46% | 62.25% | 78.48% | 53.16% | 63.38% | 79.04% | 49.88% | 61.16% |  |  |  |
|  | Clair3 | HG004 | 97.07% | 92.56% | 94.76% | 99.11% | 98.92% | 99.02% | 77.39% | 51.07% | 61.53% | 75.49% | 52.71% | 62.08% | 79.35% | 49.56% | 61.01% |  |  |  |
| 30x | Clair3 | HG002 | 97.70% | 93.33% | 95.47% | 99.39% | 99.40% | 99.40% | 82.05% | 54.86% | 65.75% | 83.51% | 55.78% | 66.89% | 80.68% | 54.01% | 64.70% | 28434 | 35 | 990 |
|  | Clair3 | HG003 | 97.82% | 93.92% | 95.83% | 99.45% | 99.51% | 99.48% | 82.57% | 57.00% | 67.44% | 82.08% | 58.75% | 68.48% | 83.07% | 55.36% | 66.44% |  |  |  |
|  | Clair3 | HG004 | 98.02% | 93.80% | 95.86% | 99.66% | 99.58% | 99.62% | 82.69% | 56.14% | 66.88% | 80.50% | 58.18% | 67.55% | 84.97% | 54.26% | 66.23% |  |  |  |
| 40x | Clair3 | HG002 | 98.28% | 93.92% | 96.05% | 99.64% | 99.67% | 99.65% | 85.73% | 57.51% | 68.84% | 86.75% | 58.44% | 69.83% | 84.78% | 56.64% | 67.91% | 30352 | 35 | 652 |
|  | Clair3 | HG003 | 98.43% | 94.41% | 96.38% | 99.62% | 99.68% | 99.65% | 87.21% | 59.65% | 70.84% | 87.49% | 61.69% | 72.36% | 86.94% | 57.74% | 69.39% |  |  |  |
|  | Clair3 | HG004 | 98.55% | 94.27% | 96.37% | 99.76% | 99.69% | 99.72% | 87.27% | 58.95% | 70.37% | 85.18% | 60.95% | 71.06% | 89.43% | 57.11% | 69.70% |  |  |  |
| 50x | Clair3 | HG002 | 98.51% | 94.31% | 96.36% | 99.73% | 99.74% | 99.73% | 87.48% | 59.89% | 71.10% | 88.04% | 60.07% | 71.41% | 86.97% | 59.72% | 70.81% | 30674 | 35 | 502 |
|  | Clair3 | HG003 | 98.60% | 94.76% | 96.64% | 99.67% | 99.70% | 99.68% | 88.81% | 62.19% | 73.15% | 89.03% | 64.38% | 74.73% | 88.59% | 60.14% | 71.64% |  |  |  |
|  | Clair3 | HG004 | 98.74% | 94.70% | 96.68% | 99.80% | 99.74% | 99.77% | 89.04% | 61.81% | 72.97% | 87.23% | 63.88% | 73.75% | 90.90% | 59.92% | 72.22% |  |  |  |
| 60x | Clair3 | HG002 | 98.51% | 94.58% | 96.50% | 99.78% | 99.74% | 99.76% | 87.41% | 61.86% | 72.45% | 88.53% | 61.86% | 72.83% | 86.39% | 61.86% | 72.10% | 30725 | 35 | 458 |
|  | Clair3 | HG003 | 98.67% | 95.10% | 96.85% | 99.70% | 99.73% | 99.71% | 89.51% | 64.57% | 75.02% | 90.06% | 66.06% | 76.21% | 88.96% | 63.18% | 73.89% |  |  |  |
|  | Clair3 | HG004 | 98.88% | 95.08% | 96.94% | 99.81% | 99.76% | 99.79% | 90.60% | 64.56% | 75.39% | 89.38% | 66.95% | 76.56% | 91.84% | 62.36% | 74.28% |  |  |  |
| 70x | Clair3 | HG002 | 98.53% | 94.87% | 96.67% | 99.79% | 99.76% | 99.77% | 87.86% | 63.88% | 73.97% | 89.66% | 63.59% | 74.41% | 86.27% | 64.14% | 73.58% | 32041 | 35 | 428 |
|  | Clair3 | HG003 | 98.66% | 95.32% | 96.96% | 99.71% | 99.74% | 99.72% | 89.54% | 66.13% | 76.08% | 90.62% | 67.23% | 77.19% | 88.52% | 65.11% | 75.03% |  |  |  |
|  | Clair3 | HG004 | 98.94% | 95.29% | 97.08% | 99.84% | 99.78% | 99.81% | 91.08% | 66.08% | 76.59% | 90.04% | 68.34% | 77.70% | 92.12% | 64.02% | 75.54% |  |  |  |
| 80x | Clair3 | HG002 | 98.47% | 95.05% | 96.73% | 99.81% | 99.78% | 99.79% | 87.41% | 65.04% | 74.58% | 90.02% | 64.55% | 75.19% | 85.14% | 65.49% | 74.04% | 31996 | 35 | 438 |
|  | Clair3 | HG003 | 98.68% | 95.47% | 97.05% | 99.71% | 99.74% | 99.72% | 89.79% | 67.31% | 76.94% | 91.30% | 67.93% | 77.90% | 88.39% | 66.73% | 76.05% |  |  |  |
|  | Clair3 | HG004 | 99.01% | 95.51% | 97.23% | 99.85% | 99.80% | 99.82% | 91.87% | 67.61% | 77.90% | 91.00% | 69.63% | 78.89% | 92.74% | 65.76% | 76.95% |  |  |  |
| 90x | Clair3 | HG002 | 98.53% | 95.15% | 96.81% | 99.84% | 99.79% | 99.82% | 87.67% | 65.72% | 75.12% | 90.66% | 64.76% | 75.55% | 85.12% | 66.60% | 74.73% | 32368 | 35 | 420 |
|  | Clair3 | HG003 | 98.74% | 95.49% | 97.08% | 99.72% | 99.73% | 99.72% | 90.24% | 67.51% | 77.24% | 91.82% | 68.39% | 78.39% | 88.77% | 66.69% | 76.16% |  |  |  |
|  | Clair3 | HG004 | 99.05% | 95.62% | 97.30% | 99.86% | 99.81% | 99.83% | 92.16% | 68.28% | 78.45% | 91.47% | 70.10% | 79.37% | 92.84% | 66.61% | 77.57% |  |  |  |
| 10x | PEPPER | HG002 | 41.37% | 81.04% | 54.77% | 41.61% | 89.10% | 56.73% | 37.28% | 29.92% | 33.20% | 32.93% | 32.90% | 32.91% | 43.72% | 27.17% | 33.51% | 131509 | 31 | 87600 |
|  | PEPPER | HG003 | 54.01% | 83.86% | 65.70% | 54.89% | 91.51% | 68.62% | 41.90% | 33.37% | 37.15% | 37.34% | 37.35% | 37.35% | 48.92% | 29.65% | 36.92% |  |  |  |
|  | PEPPER | HG004 | 50.55% | 83.40% | 62.95% | 51.21% | 91.22% | 65.60% | 40.95% | 32.50% | 36.24% | 35.56% | 36.33% | 35.94% | 49.50% | 28.97% | 36.55% |  |  |  |
| 20x | PEPPER | HG002 | 79.59% | 91.19% | 84.99% | 80.40% | 98.57% | 88.56% | 69.81% | 44.40% | 54.28% | 68.85% | 47.19% | 56.00% | 70.84% | 41.82% | 52.59% | 43023 | 32 | 17434 |
|  | PEPPER | HG003 | 87.96% | 92.13% | 90.00% | 89.30% | 98.97% | 93.89% | 73.03% | 46.95% | 57.15% | 71.07% | 51.42% | 59.67% | 75.35% | 42.77% | 54.56% |  |  |  |
|  | PEPPER | HG004 | 87.86% | 91.92% | 89.84% | 89.28% | 99.00% | 93.89% | 71.98% | 45.77% | 55.96% | 67.84% | 49.75% | 57.41% | 77.07% | 42.11% | 54.46% |  |  |  |
| 30x | PEPPER | HG002 | 95.50% | 92.58% | 94.02% | 97.02% | 99.44% | 98.21% | 79.80% | 49.08% | 60.77% | 78.99% | 52.03% | 62.74% | 80.65% | 46.34% | 58.86% | 25540 | 34 | 2551 |
|  | PEPPER | HG003 | 96.97% | 93.24% | 95.07% | 98.52% | 99.54% | 99.03% | 81.08% | 51.65% | 63.10% | 78.89% | 55.90% | 65.44% | 83.63% | 47.68% | 60.73% |  |  |  |
|  | PEPPER | HG004 | 97.14% | 93.07% | 95.06% | 98.74% | 99.60% | 99.17% | 80.69% | 50.50% | 62.12% | 76.23% | 54.35% | 63.45% | 86.04% | 46.96% | 60.76% |  |  |  |
| 40x | PEPPER | HG002 | 97.92% | 93.08% | 95.44% | 99.26% | 99.62% | 99.44% | 84.26% | 51.61% | 64.01% | 84.48% | 54.58% | 66.32% | 84.03% | 48.85% | 61.78% | 22279 | 35 | 826 |
|  | PEPPER | HG003 | 98.05% | 93.64% | 95.79% | 99.38% | 99.66% | 99.52% | 84.53% | 53.90% | 65.83% | 82.31% | 58.18% | 68.17% | 87.08% | 49.90% | 63.45% |  |  |  |
|  | PEPPER | HG004 | 98.16% | 93.47% | 95.76% | 99.56% | 99.70% | 99.63% | 84.02% | 52.90% | 64.92% | 80.30% | 56.80% | 66.53% | 88.37% | 49.31% | 63.30% |  |  |  |
| 50x | PEPPER | HG002 | 98.43% | 93.32% | 95.81% | 99.62% | 99.69% | 99.65% | 86.37% | 52.95% | 65.65% | 86.29% | 55.76% | 67.75% | 86.44% | 50.34% | 63.63% | 21267 | 35 | 524 |
|  | PEPPER | HG003 | 98.41% | 93.86% | 96.08% | 99.57% | 99.69% | 99.63% | 86.66% | 55.40% | 67.59% | 84.83% | 59.53% | 69.96% | 88.71% | 51.54% | 65.20% |  |  |  |
|  | PEPPER | HG004 | 98.50% | 93.70% | 96.04% | 99.68% | 99.73% | 99.70% | 86.54% | 54.39% | 66.80% | 82.91% | 58.30% | 68.46% | 90.74% | 50.79% | 65.13% |  |  |  |
| 60x | PEPPER | HG002 | 98.61% | 93.48% | 95.98% | 99.68% | 99.70% | 99.69% | 87.81% | 54.09% | 66.94% | 86.99% | 56.58% | 68.56% | 88.67% | 51.78% | 65.38% | 20559 | 35 | 455 |
|  | PEPPER | HG003 | 98.64% | 94.02% | 96.27% | 99.61% | 99.72% | 99.67% | 88.70% | 56.35% | 68.91% | 86.80% | 60.31% | 71.17% | 90.82% | 52.65% | 66.66% |  |  |  |
|  | PEPPER | HG004 | 98.74% | 93.89% | 96.26% | 99.76% | 99.77% | 99.77% | 88.37% | 55.61% | 68.27% | 85.10% | 59.24% | 69.86% | 92.07% | 52.28% | 66.69% |  |  |  |
| 70x | PEPPER | HG002 | 98.81% | 93.65% | 96.16% | 99.72% | 99.73% | 99.72% | 89.63% | 55.07% | 68.22% | 89.13% | 57.61% | 69.98% | 90.14% | 52.72% | 66.53% | 19583 | 35 | 359 |
|  | PEPPER | HG003 | 98.76% | 94.14% | 96.39% | 99.66% | 99.74% | 99.70% | 89.64% | 57.16% | 69.80% | 88.38% | 60.95% | 72.15% | 91.02% | 53.61% | 67.48% |  |  |  |
|  | PEPPER | HG004 | 98.84% | 93.98% | 96.35% | 99.76% | 99.79% | 99.78% | 89.51% | 56.14% | 69.00% | 86.81% | 59.66% | 70.72% | 92.49% | 52.90% | 67.31% |  |  |  |
| 80x | PEPPER | HG002 | 98.93% | 93.78% | 96.28% | 99.76% | 99.78% | 99.77% | 90.52% | 55.73% | 68.99% | 90.16% | 58.27% | 70.79% | 90.89% | 53.37% | 67.25% | 20463 | 35 | 349 |
|  | PEPPER | HG003 | 98.85% | 94.19% | 96.46% | 99.66% | 99.75% | 99.71% | 90.54% | 57.47% | 70.31% | 89.00% | 61.40% | 72.67% | 92.25% | 53.79% | 67.96% |  |  |  |
|  | PEPPER | HG004 | 98.91% | 93.98% | 96.38% | 99.79% | 99.79% | 99.79% | 89.93% | 56.12% | 69.11% | 87.42% | 59.83% | 71.04% | 92.72% | 52.71% | 67.21% |  |  |  |
| 90x | PEPPER | HG002 | 98.98% | 93.82% | 96.33% | 99.77% | 99.77% | 99.77% | 91.06% | 56.16% | 69.47% | 90.63% | 58.64% | 71.21% | 91.50% | 53.85% | 67.80% | 20373 | 35 | 321 |
|  | PEPPER | HG003 | 98.84% | 94.26% | 96.50% | 99.67% | 99.75% | 99.71% | 90.55% | 58.00% | 70.71% | 89.23% | 61.91% | 73.10% | 91.99% | 54.36% | 68.33% |  |  |  |
|  | PEPPER | HG004 | 98.90% | 94.06% | 96.42% | 99.79% | 99.82% | 99.80% | 89.97% | 56.57% | 69.46% | 87.24% | 60.14% | 71.19% | 93.00% | 53.29% | 67.75% |  |  |  |
| 10x | Clair3-Trio | HG002 | 96.71% | 89.30% | 92.85% | 98.07% | 96.55% | 97.30% | 81.05% | 43.34% | 56.48% | 80.65% | 44.05% | 56.98% | 81.44% | 42.67% | 56.00% | 7072 | 24 | 494 |
|  | Clair3-Trio | HG003 | 94.92% | 89.48% | 92.12% | 96.44% | 96.55% | 96.49% | 77.07% | 42.85% | 55.07% | 78.75% | 44.68% | 57.01% | 75.44% | 41.13% | 53.24% |  |  |  |
|  | Clair3-Trio | HG004 | 94.63% | 89.15% | 91.81% | 95.96% | 96.41% | 96.18% | 78.50% | 41.83% | 54.58% | 80.17% | 43.19% | 56.13% | 76.92% | 40.59% | 53.14% |  |  |  |
| 20x | Clair3-Trio | HG002 | 98.45% | 93.51% | 95.91% | 99.59% | 99.49% | 99.54% | 87.30% | 55.57% | 67.91% | 86.90% | 56.41% | 68.41% | 87.69% | 54.79% | 67.44% | 6243 | 24 | 188 |
|  | Clair3-Trio | HG003 | 98.23% | 93.46% | 95.79% | 99.39% | 99.42% | 99.40% | 86.30% | 54.13% | 66.53% | 86.70% | 55.46% | 67.64% | 85.91% | 52.88% | 65.47% |  |  |  |
|  | Clair3-Trio | HG004 | 98.39% | 93.35% | 95.81% | 99.46% | 99.50% | 99.48% | 87.18% | 53.33% | 66.18% | 87.22% | 54.42% | 67.02% | 87.15% | 52.33% | 65.39% |  |  |  |
| 30x | Clair3-Trio | HG002 | 98.66% | 94.34% | 96.45% | 99.73% | 99.69% | 99.71% | 88.90% | 60.43% | 71.95% | 88.29% | 61.38% | 72.41% | 89.51% | 59.55% | 71.52% | 6935 | 31 | 176 |
|  | Clair3-Trio | HG003 | 98.66% | 94.26% | 96.41% | 99.65% | 99.61% | 99.63% | 89.10% | 58.97% | 70.97% | 88.81% | 60.15% | 71.73% | 89.39% | 57.87% | 70.25% |  |  |  |
|  | Clair3-Trio | HG004 | 98.83% | 94.19% | 96.45% | 99.76% | 99.69% | 99.72% | 89.72% | 58.39% | 70.74% | 89.34% | 59.62% | 71.52% | 90.09% | 57.25% | 70.01% |  |  |  |
| 40x | Clair3-Trio | HG002 | 98.73% | 94.84% | 96.75% | 99.76% | 99.74% | 99.75% | 89.79% | 63.79% | 74.59% | 89.16% | 64.59% | 74.91% | 90.41% | 63.04% | 74.29% | 7392 | 33 | 173 |
|  | Clair3-Trio | HG003 | 98.82% | 94.86% | 96.80% | 99.69% | 99.70% | 99.69% | 90.82% | 62.91% | 74.33% | 90.69% | 64.52% | 75.40% | 90.94% | 61.42% | 73.32% |  |  |  |
|  | Clair3-Trio | HG004 | 98.83% | 94.72% | 96.73% | 99.77% | 99.75% | 99.76% | 90.11% | 61.90% | 73.39% | 90.21% | 63.36% | 74.44% | 90.01% | 60.56% | 72.41% |  |  |  |
| 50x | Clair3-Trio | HG002 | 98.69% | 95.30% | 96.96% | 99.78% | 99.79% | 99.79% | 89.64% | 66.83% | 76.57% | 88.66% | 67.58% | 76.70% | 90.60% | 66.12% | 76.45% | 7748 | 33 | 215 |
|  | Clair3-Trio | HG003 | 98.82% | 95.24% | 97.00% | 99.71% | 99.71% | 99.71% | 90.87% | 65.70% | 76.26% | 90.59% | 67.07% | 77.08% | 91.14% | 64.42% | 75.48% |  |  |  |
|  | Clair3-Trio | HG004 | 98.89% | 95.16% | 96.99% | 99.81% | 99.79% | 99.80% | 90.79% | 64.99% | 75.75% | 90.48% | 66.53% | 76.68% | 91.08% | 63.56% | 74.87% |  |  |  |
| 60x | Clair3-Trio | HG002 | 98.72% | 95.54% | 97.10% | 99.78% | 99.75% | 99.77% | 90.14% | 68.84% | 78.07% | 89.31% | 69.80% | 78.36% | 90.96% | 67.96% | 77.79% | 8429 | 33 | 197 |
|  | Clair3-Trio | HG003 | 98.81% | 95.47% | 97.11% | 99.72% | 99.72% | 99.72% | 90.90% | 67.39% | 77.40% | 90.75% | 69.25% | 78.56% | 91.06% | 65.65% | 76.30% |  |  |  |
|  | Clair3-Trio | HG004 | 98.90% | 95.43% | 97.13% | 99.81% | 99.79% | 99.80% | 91.14% | 66.98% | 77.22% | 90.85% | 68.09% | 77.84% | 91.41% | 65.97% | 76.63% |  |  |  |
| 70x | Clair3-Trio | HG002 | 98.77% | 95.73% | 97.23% | 99.79% | 99.78% | 99.78% | 90.63% | 70.06% | 79.03% | 90.15% | 71.20% | 79.57% | 91.09% | 69.00% | 78.52% | 9052 | 32 | 183 |
|  | Clair3-Trio | HG003 | 98.84% | 95.66% | 97.22% | 99.74% | 99.72% | 99.73% | 91.19% | 68.86% | 78.47% | 90.96% | 70.44% | 79.40% | 91.42% | 67.38% | 77.58% |  |  |  |
|  | Clair3-Trio | HG004 | 98.94% | 95.57% | 97.22% | 99.80% | 99.80% | 99.80% | 91.55% | 68.01% | 78.04% | 91.09% | 69.23% | 78.67% | 91.99% | 66.89% | 77.46% |  |  |  |
| 80x | Clair3-Trio | HG002 | 98.79% | 95.85% | 97.29% | 99.79% | 99.81% | 99.80% | 90.79% | 70.75% | 79.53% | 90.16% | 71.72% | 79.89% | 91.41% | 69.86% | 79.19% | 9096 | 33 | 185 |
|  | Clair3-Trio | HG003 | 98.86% | 95.77% | 97.29% | 99.72% | 99.75% | 99.73% | 91.64% | 69.55% | 79.08% | 91.33% | 71.06% | 79.93% | 91.94% | 68.13% | 78.26% |  |  |  |
|  | Clair3-Trio | HG004 | 99.04% | 95.70% | 97.34% | 99.81% | 99.80% | 99.81% | 92.47% | 68.95% | 78.99% | 92.28% | 70.24% | 79.76% | 92.65% | 67.77% | 78.28% |  |  |  |
| 90x | Clair3-Trio | HG002 | 98.78% | 95.91% | 97.33% | 99.80% | 99.82% | 99.81% | 90.74% | 71.17% | 79.77% | 90.23% | 72.02% | 80.10% | 91.23% | 70.39% | 79.46% | 9076 | 32 | 198 |
|  | Clair3-Trio | HG003 | 98.93% | 95.84% | 97.36% | 99.76% | 99.75% | 99.76% | 91.85% | 70.00% | 79.45% | 92.25% | 71.82% | 80.76% | 91.45% | 68.29% | 78.19% |  |  |  |
|  | Clair3-Trio | HG004 | 99.03% | 95.70% | 97.33% | 99.83% | 99.81% | 99.82% | 92.28% | 68.92% | 78.91% | 92.33% | 69.97% | 79.61% | 92.22% | 67.96% | 78.25% |  |  |  |

## Supplementary Table 2. HG002 trio’s *de novo* variants failed to be detected by Clair3-Trio.

| CHR | POS | REF | ALT | found by Clair3 | found by PEPPER | found by Clair3-Trio |
| --- | --- | --- | --- | --- | --- | --- |
| chr20 | 3390371 | CT | C | N | N | N |
| chr20 | 18487996 | C | G | Y | Y | N |
| chr20 | 36652410 | C | CA | N | N | N |
| chr20 | 43658577 | A | C | Y | Y | N |
| chr20 | 47138749 | CA | C | N | N | N |
| chr20 | 51068541 | T | TA | N | N | N |
| chr20 | 57665372 | C | TA | N | N | N |

## Supplementary Table 3. HG005 trio benchmarking results.

| Tool | Sample | Coverage | Overall | | | SNP | | | Indel | | | Insertion | | | Deletion | | | # of MCV | *de novo* TP | *de novo* FP |
| --- | --- | --- | --- | --- | --- | --- | --- | --- | --- | --- | --- | --- | --- | --- | --- | --- | --- | --- | --- | --- |
|  |  |  | Precision | Recall | F1-Score | Precision | Recall | F1-Score | Precision | Recall | F1-Score | Precision | Recall | F1-Score | Precision | Recall | F1-Score |  |  |  |
| Clair3-Trio | HG005 | 40x | 98.94% | 97.40% | 98.16% | 99.74% | 99.88% | 99.81% | 91.49% | 77.25% | 83.77% | 91.19% | 76.68% | 83.31% | 91.77% | 77.79% | 84.20% | 7056 | 0 | 106 |
| Clair3-Trio | HG006 | 40x | 98.86% | 96.34% | 97.58% | 99.70% | 99.84% | 99.77% | 90.61% | 69.35% | 78.57% | 89.86% | 68.32% | 77.62% | 91.34% | 70.38% | 79.50% |  |  |  |
| Clair3-Trio | HG007 | 40x | 99.01% | 96.16% | 97.57% | 99.83% | 99.80% | 99.81% | 90.89% | 68.23% | 77.95% | 91.97% | 67.43% | 77.81% | 89.86% | 69.03% | 78.08% |  |  |  |
| Clair3 | HG005 | 40x | 98.75% | 96.48% | 97.60% | 99.72% | 99.74% | 99.73% | 88.97% | 70.00% | 78.35% | 90.19% | 70.69% | 79.26% | 87.85% | 69.35% | 77.51% | 18723 | 0 | 399 |
| Clair3 | HG006 | 40x | 98.81% | 96.01% | 97.39% | 99.72% | 99.80% | 99.76% | 89.67% | 66.77% | 76.54% | 88.93% | 66.35% | 76.00% | 90.40% | 67.19% | 77.09% |  |  |  |
| Clair3 | HG007 | 40x | 98.81% | 95.79% | 97.28% | 99.83% | 99.72% | 99.77% | 88.52% | 65.59% | 75.35% | 89.97% | 65.41% | 75.75% | 87.13% | 65.76% | 74.95% |  |  |  |
| PEPPER | HG005 | 40x | 98.34% | 95.73% | 97.02% | 99.42% | 99.75% | 99.59% | 86.43% | 63.13% | 72.97% | 86.69% | 65.65% | 74.72% | 86.16% | 60.77% | 71.27% | 19320 | 0 | 599 |
| PEPPER | HG006 | 40x | 98.46% | 95.21% | 96.81% | 99.52% | 99.79% | 99.66% | 86.72% | 59.89% | 70.85% | 84.64% | 61.96% | 71.54% | 89.04% | 57.84% | 70.12% |  |  |  |
| PEPPER | HG007 | 40x | 98.50% | 95.13% | 96.79% | 99.51% | 99.73% | 99.62% | 87.36% | 59.83% | 71.02% | 86.59% | 61.97% | 72.24% | 88.19% | 57.71% | 69.77% |  |  |  |

## Supplementary Table 4. HG002 trio multiple-coverage benchmarking results at Guppy5 data.

The Guppy5 data are basecalled with Guppy5 using the dna_r9.4.1_450bps_sup profile with fast5 raw data obtained from the Human Pangenome Reference Consortium (HPRC). The full coverage of the three samples are HG002 (~117.4x), HG003 (~78.9x), and HG004 (~79.0x). For Clair3, we used v0.1-r11 and the r941_prom_sup_g5014 model. For PEPPER, we used r0.8 and the ont_r9_guppy5_sup model. “80x” means up to 80x in the table.

| Coverage | Tool | Sample | Overall | | | SNP | | | Indel | | | Insertion | | | Deletion | | | # of MCV | *de novo* TP | *de novo* FP |
| --- | --- | --- | --- | --- | --- | --- | --- | --- | --- | --- | --- | --- | --- | --- | --- | --- | --- | --- | --- | --- |
|  |  |  | Precision | Recall | F1-Score | Precision | Recall | F1-Score | Precision | Recall | F1-Score | Precision | Recall | F1-Score | Precision | Recall | F1-Score |  |  |  |
| 10x | Clair3 | HG002 | 94.57% | 87.31% | 90.79% | 95.74% | 94.33% | 95.03% | 80.90% | 42.77% | 55.95% | 81.05% | 43.90% | 56.96% | 80.74% | 41.72% | 55.01% | 38425 | 33 | 3503 |
|  | Clair3 | HG003 | 95.32% | 88.62% | 91.85% | 96.49% | 95.38% | 95.93% | 81.46% | 43.99% | 57.13% | 83.36% | 45.50% | 58.87% | 79.63% | 42.59% | 55.50% |  |  |  |
|  | Clair3 | HG004 | 95.44% | 88.40% | 91.79% | 96.57% | 95.34% | 95.95% | 81.91% | 43.17% | 56.54% | 82.64% | 44.55% | 57.89% | 81.21% | 41.90% | 55.28% |  |  |  |
| 20x | Clair3 | HG002 | 98.04% | 93.56% | 95.75% | 99.38% | 99.37% | 99.38% | 85.40% | 56.71% | 68.16% | 84.85% | 57.24% | 68.36% | 85.93% | 56.21% | 67.97% | 34283 | 35 | 816 |
|  | Clair3 | HG003 | 98.24% | 93.85% | 95.99% | 99.41% | 99.33% | 99.37% | 86.81% | 57.64% | 69.28% | 86.78% | 59.04% | 70.27% | 86.83% | 56.34% | 68.34% |  |  |  |
|  | Clair3 | HG004 | 98.37% | 93.83% | 96.05% | 99.56% | 99.45% | 99.51% | 86.85% | 57.23% | 69.00% | 86.99% | 58.03% | 69.62% | 86.71% | 56.50% | 68.42% |  |  |  |
| 30x | Clair3 | HG002 | 98.49% | 94.78% | 96.60% | 99.67% | 99.80% | 99.74% | 88.14% | 62.98% | 73.47% | 87.67% | 64.31% | 74.20% | 88.60% | 61.74% | 72.77% | 38068 | 36 | 554 |
|  | Clair3 | HG003 | 98.64% | 95.10% | 96.83% | 99.62% | 99.75% | 99.68% | 89.79% | 64.38% | 74.99% | 89.16% | 65.74% | 75.68% | 90.42% | 63.11% | 74.33% |  |  |  |
|  | Clair3 | HG004 | 98.71% | 94.94% | 96.79% | 99.76% | 99.76% | 99.76% | 89.33% | 63.53% | 74.25% | 89.28% | 64.37% | 74.80% | 89.38% | 62.76% | 73.74% |  |  |  |
| 40x | Clair3 | HG002 | 98.68% | 95.31% | 96.96% | 99.76% | 99.83% | 99.80% | 89.64% | 66.60% | 76.43% | 88.58% | 68.43% | 77.22% | 90.71% | 64.91% | 75.67% | 40964 | 35 | 505 |
|  | Clair3 | HG003 | 98.73% | 95.60% | 97.14% | 99.65% | 99.79% | 99.72% | 90.76% | 67.94% | 77.71% | 90.00% | 69.64% | 78.52% | 91.52% | 66.35% | 76.93% |  |  |  |
|  | Clair3 | HG004 | 98.86% | 95.54% | 97.17% | 99.82% | 99.83% | 99.82% | 90.75% | 67.54% | 77.44% | 90.02% | 68.58% | 77.85% | 91.46% | 66.58% | 77.06% |  |  |  |
| 50x | Clair3 | HG002 | 98.77% | 95.75% | 97.24% | 99.81% | 99.86% | 99.84% | 90.36% | 69.71% | 78.70% | 88.66% | 71.35% | 79.07% | 92.07% | 68.20% | 78.36% | 40633 | 35 | 474 |
|  | Clair3 | HG003 | 98.79% | 95.99% | 97.37% | 99.68% | 99.81% | 99.75% | 91.34% | 70.75% | 79.74% | 90.23% | 73.09% | 80.76% | 92.47% | 68.56% | 78.74% |  |  |  |
|  | Clair3 | HG004 | 98.80% | 95.92% | 97.34% | 99.83% | 99.84% | 99.83% | 90.44% | 70.35% | 79.14% | 89.85% | 71.66% | 79.73% | 91.00% | 69.14% | 78.58% |  |  |  |
| 60x | Clair3 | HG002 | 98.78% | 95.98% | 97.36% | 99.82% | 99.87% | 99.85% | 90.56% | 71.37% | 79.82% | 88.44% | 73.42% | 80.23% | 92.73% | 69.46% | 79.43% | 41248 | 35 | 440 |
|  | Clair3 | HG003 | 98.80% | 96.25% | 97.51% | 99.71% | 99.81% | 99.76% | 91.40% | 72.74% | 81.01% | 90.14% | 75.12% | 81.95% | 92.70% | 70.53% | 80.11% |  |  |  |
|  | Clair3 | HG004 | 98.90% | 96.19% | 97.53% | 99.84% | 99.86% | 99.85% | 91.41% | 72.30% | 80.74% | 90.41% | 73.80% | 81.27% | 92.39% | 70.92% | 80.25% |  |  |  |
| 70x | Clair3 | HG002 | 98.76% | 96.18% | 97.45% | 99.82% | 99.87% | 99.84% | 90.57% | 72.81% | 80.73% | 88.74% | 74.53% | 81.02% | 92.43% | 71.23% | 80.45% | 41812 | 35 | 417 |
|  | Clair3 | HG003 | 98.79% | 96.47% | 97.62% | 99.71% | 99.80% | 99.76% | 91.51% | 74.52% | 82.15% | 90.30% | 76.99% | 83.11% | 92.75% | 72.22% | 81.21% |  |  |  |
|  | Clair3 | HG004 | 98.92% | 96.39% | 97.64% | 99.87% | 99.87% | 99.87% | 91.44% | 73.72% | 81.63% | 90.61% | 75.09% | 82.13% | 92.26% | 72.46% | 81.17% |  |  |  |
| 80x | Clair3 | HG002 | 98.86% | 96.42% | 97.63% | 99.82% | 99.87% | 99.85% | 91.53% | 74.56% | 82.18% | 89.86% | 76.27% | 82.51% | 93.21% | 72.97% | 81.86% | 42606 | 36 | 393 |
|  | Clair3 | HG003 | 98.81% | 96.65% | 97.72% | 99.72% | 99.82% | 99.77% | 91.69% | 75.67% | 82.91% | 90.25% | 78.16% | 83.77% | 93.17% | 73.35% | 82.08% |  |  |  |
|  | Clair3 | HG004 | 98.93% | 96.58% | 97.74% | 99.87% | 99.87% | 99.87% | 91.70% | 75.10% | 82.58% | 90.89% | 76.37% | 83.00% | 92.49% | 73.94% | 82.18% |  |  |  |
| 10x | PEPPER | HG002 | 96.53% | 80.09% | 87.55% | 97.61% | 86.86% | 91.92% | 83.05% | 37.19% | 51.37% | 84.32% | 39.25% | 53.57% | 81.78% | 35.28% | 49.29% | 32378 | 32 | 3512 |
|  | PEPPER | HG003 | 96.74% | 82.11% | 88.83% | 97.87% | 88.64% | 93.03% | 82.65% | 39.04% | 53.04% | 85.69% | 41.80% | 56.19% | 79.64% | 36.47% | 50.03% |  |  |  |
|  | PEPPER | HG004 | 96.79% | 81.84% | 88.69% | 97.88% | 88.51% | 92.96% | 83.10% | 38.38% | 52.51% | 85.89% | 40.62% | 55.16% | 80.42% | 36.32% | 50.04% |  |  |  |
| 20x | PEPPER | HG002 | 98.11% | 92.98% | 95.47% | 99.68% | 98.60% | 99.14% | 83.97% | 57.30% | 68.12% | 85.63% | 57.55% | 68.84% | 82.47% | 57.07% | 67.46% | 25142 | 35 | 797 |
|  | PEPPER | HG003 | 98.24% | 93.50% | 95.81% | 99.69% | 98.74% | 99.21% | 84.93% | 58.88% | 69.54% | 87.95% | 59.61% | 71.05% | 82.24% | 58.20% | 68.16% |  |  |  |
|  | PEPPER | HG004 | 98.20% | 93.42% | 95.75% | 99.78% | 98.85% | 99.31% | 83.88% | 58.02% | 68.59% | 86.94% | 57.97% | 69.56% | 81.25% | 58.07% | 67.73% |  |  |  |
| 30x | PEPPER | HG002 | 98.31% | 94.86% | 96.55% | 99.79% | 99.62% | 99.70% | 86.13% | 64.71% | 73.90% | 87.88% | 64.52% | 74.41% | 84.59% | 64.89% | 73.44% | 24903 | 36 | 542 |
|  | PEPPER | HG003 | 98.25% | 95.26% | 96.73% | 99.72% | 99.61% | 99.67% | 86.03% | 66.57% | 75.06% | 88.83% | 66.25% | 75.90% | 83.59% | 66.87% | 74.30% |  |  |  |
|  | PEPPER | HG004 | 98.36% | 94.91% | 96.60% | 99.88% | 99.61% | 99.74% | 85.60% | 64.29% | 73.43% | 88.49% | 63.34% | 73.83% | 83.18% | 65.15% | 73.07% |  |  |  |
| 40x | PEPPER | HG002 | 98.42% | 95.55% | 96.96% | 99.84% | 99.73% | 99.78% | 87.34% | 69.03% | 77.11% | 88.66% | 68.40% | 77.22% | 86.18% | 69.62% | 77.02% | 26250 | 36 | 483 |
|  | PEPPER | HG003 | 98.28% | 95.93% | 97.09% | 99.73% | 99.74% | 99.73% | 86.85% | 70.82% | 78.02% | 89.47% | 70.71% | 78.99% | 84.55% | 70.91% | 77.13% |  |  |  |
|  | PEPPER | HG004 | 98.39% | 95.71% | 97.03% | 99.89% | 99.77% | 99.83% | 86.60% | 69.25% | 76.96% | 88.97% | 67.75% | 76.92% | 84.61% | 70.63% | 76.99% |  |  |  |
| 50x | PEPPER | HG002 | 98.49% | 96.02% | 97.24% | 99.85% | 99.80% | 99.82% | 88.19% | 72.02% | 79.29% | 89.00% | 71.28% | 79.16% | 87.46% | 72.71% | 79.41% | 26334 | 35 | 451 |
|  | PEPPER | HG003 | 98.43% | 96.36% | 97.39% | 99.74% | 99.77% | 99.75% | 88.39% | 73.85% | 80.47% | 90.61% | 73.46% | 81.14% | 86.44% | 74.20% | 79.86% |  |  |  |
|  | PEPPER | HG004 | 98.48% | 96.14% | 97.30% | 99.90% | 99.81% | 99.86% | 87.56% | 72.25% | 79.17% | 89.77% | 70.65% | 79.07% | 85.70% | 73.71% | 79.26% |  |  |  |
| 60x | PEPPER | HG002 | 98.53% | 96.30% | 97.40% | 99.86% | 99.82% | 99.84% | 88.71% | 73.96% | 80.67% | 88.92% | 73.46% | 80.45% | 88.53% | 74.43% | 80.87% | 27110 | 36 | 401 |
|  | PEPPER | HG003 | 98.47% | 96.70% | 97.58% | 99.75% | 99.79% | 99.77% | 88.90% | 76.31% | 82.12% | 91.00% | 75.94% | 82.79% | 87.04% | 76.66% | 81.52% |  |  |  |
|  | PEPPER | HG004 | 98.61% | 96.46% | 97.53% | 99.91% | 99.85% | 99.88% | 88.82% | 74.41% | 80.98% | 91.01% | 73.04% | 81.05% | 86.96% | 75.67% | 80.92% |  |  |  |
| 70x | PEPPER | HG002 | 98.57% | 96.60% | 97.58% | 99.87% | 99.85% | 99.86% | 89.16% | 76.00% | 82.06% | 89.59% | 75.71% | 82.07% | 88.77% | 76.28% | 82.05% | 27481 | 36 | 404 |
|  | PEPPER | HG003 | 98.51% | 96.90% | 97.70% | 99.74% | 99.81% | 99.78% | 89.37% | 77.71% | 83.13% | 90.85% | 77.32% | 83.54% | 88.05% | 78.08% | 82.77% |  |  |  |
|  | PEPPER | HG004 | 98.73% | 96.67% | 97.69% | 99.92% | 99.83% | 99.88% | 89.86% | 76.10% | 82.41% | 91.31% | 74.75% | 82.21% | 88.60% | 77.34% | 82.59% |  |  |  |
| 80x | PEPPER | HG002 | 98.64% | 96.82% | 97.72% | 99.86% | 99.85% | 99.85% | 89.88% | 77.62% | 83.30% | 89.70% | 77.67% | 83.25% | 90.06% | 77.58% | 83.35% | 28229 | 36 | 402 |
|  | PEPPER | HG003 | 98.57% | 97.10% | 97.83% | 99.76% | 99.81% | 99.78% | 89.89% | 79.19% | 84.20% | 91.10% | 79.11% | 84.69% | 88.79% | 79.26% | 83.75% |  |  |  |
|  | PEPPER | HG004 | 98.78% | 96.86% | 97.81% | 99.93% | 99.85% | 99.89% | 90.34% | 77.40% | 83.37% | 91.99% | 76.06% | 83.27% | 88.91% | 78.63% | 83.46% |  |  |  |
| 10x | Clair3-Trio | HG002 | 97.62% | 90.83% | 94.10% | 98.73% | 97.21% | 97.97% | 85.95% | 50.34% | 63.49% | 85.19% | 51.11% | 63.89% | 86.70% | 49.62% | 63.12% | 9372 | 27 | 552 |
|  | Clair3-Trio | HG003 | 96.43% | 90.57% | 93.41% | 97.52% | 96.87% | 97.20% | 84.31% | 48.97% | 61.95% | 84.77% | 50.47% | 63.27% | 83.86% | 47.57% | 60.70% |  |  |  |
|  | Clair3-Trio | HG004 | 96.55% | 90.56% | 93.46% | 97.58% | 97.18% | 97.38% | 84.81% | 47.36% | 60.78% | 84.54% | 48.54% | 61.67% | 85.08% | 46.28% | 59.95% |  |  |  |
| 20x | Clair3-Trio | HG002 | 98.51% | 94.66% | 96.55% | 99.68% | 99.67% | 99.67% | 88.27% | 62.96% | 73.50% | 87.85% | 63.48% | 73.71% | 88.68% | 62.48% | 73.31% | 8874 | 29 | 241 |
|  | Clair3-Trio | HG003 | 98.50% | 94.35% | 96.38% | 99.50% | 99.50% | 99.50% | 88.95% | 60.33% | 71.89% | 88.69% | 61.42% | 72.58% | 89.20% | 59.31% | 71.24% |  |  |  |
|  | Clair3-Trio | HG004 | 98.63% | 94.34% | 96.44% | 99.67% | 99.65% | 99.66% | 88.80% | 59.79% | 71.46% | 88.17% | 60.84% | 72.00% | 89.42% | 58.82% | 70.96% |  |  |  |
| 30x | Clair3-Trio | HG002 | 98.54% | 95.52% | 97.01% | 99.78% | 99.77% | 99.77% | 88.56% | 68.63% | 77.33% | 87.38% | 68.69% | 76.92% | 89.68% | 68.57% | 77.72% | 10562 | 30 | 261 |
|  | Clair3-Trio | HG003 | 98.65% | 95.41% | 97.00% | 99.70% | 99.74% | 99.72% | 89.57% | 66.83% | 76.54% | 88.41% | 67.15% | 76.33% | 90.70% | 66.53% | 76.75% |  |  |  |
|  | Clair3-Trio | HG004 | 98.81% | 95.29% | 97.02% | 99.82% | 99.79% | 99.80% | 90.03% | 65.99% | 76.16% | 89.27% | 66.82% | 76.43% | 90.77% | 65.24% | 75.91% |  |  |  |
| 40x | Clair3-Trio | HG002 | 98.64% | 96.08% | 97.34% | 99.81% | 99.83% | 99.82% | 89.65% | 72.27% | 80.03% | 88.32% | 72.28% | 79.50% | 90.92% | 72.27% | 80.53% | 10066 | 30 | 195 |
|  | Clair3-Trio | HG003 | 98.69% | 95.99% | 97.32% | 99.73% | 99.79% | 99.76% | 90.16% | 70.88% | 79.37% | 89.47% | 71.38% | 79.40% | 90.84% | 70.42% | 79.34% |  |  |  |
|  | Clair3-Trio | HG004 | 98.85% | 95.83% | 97.32% | 99.81% | 99.81% | 99.81% | 90.89% | 69.92% | 79.04% | 90.11% | 70.29% | 78.98% | 91.63% | 69.58% | 79.10% |  |  |  |
| 50x | Clair3-Trio | HG002 | 98.63% | 96.48% | 97.54% | 99.82% | 99.87% | 99.85% | 89.77% | 75.00% | 81.72% | 88.93% | 75.19% | 81.49% | 90.57% | 74.82% | 81.94% | 9607 | 32 | 194 |
|  | Clair3-Trio | HG003 | 98.68% | 96.37% | 97.52% | 99.74% | 99.83% | 99.78% | 90.31% | 73.55% | 81.08% | 89.83% | 74.42% | 81.40% | 90.79% | 72.75% | 80.77% |  |  |  |
|  | Clair3-Trio | HG004 | 98.80% | 96.24% | 97.50% | 99.83% | 99.83% | 99.83% | 90.70% | 72.84% | 80.79% | 90.01% | 73.48% | 80.91% | 91.35% | 72.25% | 80.68% |  |  |  |
| 60x | Clair3-Trio | HG002 | 98.66% | 96.72% | 97.68% | 99.81% | 99.86% | 99.84% | 90.24% | 76.82% | 82.99% | 89.50% | 77.06% | 82.82% | 90.95% | 76.60% | 83.16% | 9061 | 32 | 150 |
|  | Clair3-Trio | HG003 | 98.71% | 96.71% | 97.70% | 99.73% | 99.85% | 99.79% | 90.79% | 75.95% | 82.71% | 90.05% | 76.64% | 82.81% | 91.51% | 75.31% | 82.63% |  |  |  |
|  | Clair3-Trio | HG004 | 98.78% | 96.54% | 97.65% | 99.83% | 99.86% | 99.85% | 90.76% | 74.91% | 82.08% | 90.20% | 75.36% | 82.12% | 91.30% | 74.50% | 82.05% |  |  |  |
| 70x | Clair3-Trio | HG002 | 98.67% | 96.89% | 97.77% | 99.82% | 99.86% | 99.84% | 90.37% | 78.04% | 83.75% | 89.66% | 77.89% | 83.36% | 91.04% | 78.18% | 84.12% | 9258 | 34 | 170 |
|  | Clair3-Trio | HG003 | 98.77% | 96.82% | 97.79% | 99.75% | 99.79% | 99.77% | 91.29% | 77.25% | 83.69% | 90.53% | 77.84% | 83.71% | 92.03% | 76.70% | 83.67% |  |  |  |
|  | Clair3-Trio | HG004 | 98.85% | 96.74% | 97.78% | 99.85% | 99.85% | 99.85% | 91.26% | 76.43% | 83.19% | 90.58% | 76.80% | 83.12% | 91.92% | 76.09% | 83.26% |  |  |  |
| 80x | Clair3-Trio | HG002 | 98.73% | 97.10% | 97.90% | 99.83% | 99.87% | 99.85% | 90.88% | 79.50% | 84.81% | 90.51% | 79.70% | 84.76% | 91.23% | 79.32% | 84.86% | 8843 | 34 | 159 |
|  | Clair3-Trio | HG003 | 98.81% | 96.99% | 97.89% | 99.76% | 99.84% | 99.80% | 91.61% | 78.14% | 84.34% | 91.18% | 78.98% | 84.64% | 92.03% | 77.37% | 84.06% |  |  |  |
|  | Clair3-Trio | HG004 | 98.87% | 96.87% | 97.86% | 99.85% | 99.86% | 99.86% | 91.48% | 77.41% | 83.86% | 91.03% | 77.90% | 83.96% | 91.92% | 76.96% | 83.77% |  |  |  |

## Supplementary Table 5. HG002 trio multiple-coverage benchmarking results while child’s coverage fixed at 60x.

| Tool | Sample | Coverage | Overall | | | SNP | | | INDEL | | | Insertion | | | Deletion | | | # of MCV | *de novo* TP | *de novo* FP |
| --- | --- | --- | --- | --- | --- | --- | --- | --- | --- | --- | --- | --- | --- | --- | --- | --- | --- | --- | --- | --- |
|  |  |  | Precision | Recall | F1-Score | Precision | Recall | F1-Score | Precision | Recall | F1-Score | Precision | Recall | F1-Score | Precision | Recall | F1-Score |  |  |  |
| Clair3-Trio | HG002 | 60x | 98.63% | 94.43% | 96.49% | 99.70% | 99.65% | 99.67% | 89.02% | 61.37% | 72.66% | 88.22% | 62.34% | 73.06% | 89.81% | 60.48% | 72.28% | 10389 | 33 | 972 |
| Clair3-Trio | HG003 | 10x | 95.67% | 90.19% | 92.85% | 96.98% | 96.72% | 96.85% | 81.05% | 47.07% | 59.55% | 81.12% | 48.87% | 60.99% | 80.98% | 45.39% | 58.17% |  |  |  |
| Clair3-Trio | HG004 | 10x | 95.41% | 89.93% | 92.59% | 96.60% | 96.65% | 96.62% | 81.89% | 46.19% | 59.06% | 81.67% | 47.32% | 59.92% | 82.10% | 45.14% | 58.25% |  |  |  |
| Clair3-Trio | HG002 | 60x | 98.64% | 94.94% | 96.75% | 99.72% | 99.74% | 99.73% | 89.38% | 64.48% | 74.91% | 88.79% | 65.40% | 75.32% | 89.95% | 63.63% | 74.53% | 7437 | 33 | 406 |
| Clair3-Trio | HG003 | 20x | 98.34% | 93.76% | 96.00% | 99.41% | 99.44% | 99.43% | 87.48% | 56.29% | 68.50% | 87.54% | 57.95% | 69.74% | 87.41% | 54.74% | 67.32% |  |  |  |
| Clair3-Trio | HG004 | 20x | 98.48% | 93.70% | 96.03% | 99.53% | 99.52% | 99.52% | 87.95% | 55.84% | 68.31% | 87.48% | 57.02% | 69.04% | 88.41% | 54.75% | 67.63% |  |  |  |
| Clair3-Trio | HG002 | 60x | 98.72% | 95.18% | 96.92% | 99.76% | 99.76% | 99.76% | 89.97% | 66.16% | 76.25% | 89.36% | 66.97% | 76.57% | 90.55% | 65.41% | 75.95% | 7921 | 33 | 315 |
| Clair3-Trio | HG003 | 30x | 98.71% | 94.47% | 96.54% | 99.66% | 99.62% | 99.64% | 89.59% | 60.53% | 72.25% | 89.21% | 62.12% | 73.24% | 89.97% | 59.05% | 71.30% |  |  |  |
| Clair3-Trio | HG004 | 30x | 98.81% | 94.39% | 96.55% | 99.77% | 99.71% | 99.74% | 89.68% | 59.77% | 71.73% | 89.14% | 61.12% | 72.52% | 90.21% | 58.52% | 70.99% |  |  |  |
| Clair3-Trio | HG002 | 60x | 98.74% | 95.34% | 97.01% | 99.77% | 99.78% | 99.78% | 90.19% | 67.17% | 77.00% | 89.46% | 68.14% | 77.36% | 90.91% | 66.28% | 76.66% | 7860 | 32 | 248 |
| Clair3-Trio | HG003 | 40x | 98.79% | 94.95% | 96.84% | 99.69% | 99.71% | 99.70% | 90.56% | 63.56% | 74.70% | 90.18% | 65.34% | 75.77% | 90.93% | 61.91% | 73.66% |  |  |  |
| Clair3-Trio | HG004 | 40x | 98.82% | 94.82% | 96.78% | 99.79% | 99.75% | 99.77% | 89.97% | 62.67% | 73.88% | 90.02% | 64.20% | 74.95% | 89.93% | 61.26% | 72.88% |  |  |  |
| Clair3-Trio | HG002 | 60x | 98.73% | 95.45% | 97.06% | 99.79% | 99.76% | 99.78% | 90.06% | 68.10% | 77.55% | 89.22% | 69.14% | 77.90% | 90.88% | 67.13% | 77.22% | 7869 | 33 | 206 |
| Clair3-Trio | HG003 | 50x | 98.80% | 95.24% | 96.99% | 99.71% | 99.70% | 99.70% | 90.69% | 65.84% | 76.29% | 90.22% | 67.40% | 77.16% | 91.17% | 64.38% | 75.47% |  |  |  |
| Clair3-Trio | HG004 | 50x | 98.87% | 95.21% | 97.00% | 99.80% | 99.79% | 99.79% | 90.68% | 65.38% | 75.98% | 90.32% | 66.86% | 76.84% | 91.03% | 64.02% | 75.17% |  |  |  |
| Clair3-Trio | HG002 | 60x | 98.72% | 95.54% | 97.10% | 99.78% | 99.75% | 99.77% | 90.14% | 68.84% | 78.07% | 89.31% | 69.80% | 78.36% | 90.96% | 67.96% | 77.79% | 8429 | 33 | 197 |
| Clair3-Trio | HG003 | 60x | 98.81% | 95.47% | 97.11% | 99.72% | 99.72% | 99.72% | 90.90% | 67.39% | 77.40% | 90.75% | 69.25% | 78.56% | 91.06% | 65.65% | 76.30% |  |  |  |
| Clair3-Trio | HG004 | 60x | 98.90% | 95.43% | 97.13% | 99.81% | 99.79% | 99.80% | 91.14% | 66.98% | 77.22% | 90.85% | 68.09% | 77.84% | 91.41% | 65.97% | 76.63% |  |  |  |
| Clair3 | HG002 | 60x | 98.51% | 94.58% | 96.50% | 99.78% | 99.74% | 99.76% | 87.41% | 61.86% | 72.45% | 88.53% | 61.86% | 72.83% | 86.39% | 61.86% | 72.10% | 43205 | 35 | 3312 |
| Clair3 | HG003 | 10x | 87.95% | 85.63% | 86.77% | 89.59% | 92.96% | 91.24% | 67.83% | 37.24% | 48.08% | 70.41% | 39.19% | 50.35% | 65.34% | 35.42% | 45.94% |  |  |  |
| Clair3 | HG004 | 10x | 86.98% | 84.91% | 85.93% | 88.70% | 92.40% | 90.51% | 66.02% | 36.14% | 46.71% | 66.12% | 37.47% | 47.83% | 65.92% | 34.92% | 45.66% |  |  |  |
| Clair3 | HG002 | 60x | 98.51% | 94.58% | 96.50% | 99.78% | 99.74% | 99.76% | 87.41% | 61.86% | 72.45% | 88.53% | 61.86% | 72.83% | 86.39% | 61.86% | 72.10% | 33565 | 35 | 1029 |
| Clair3 | HG003 | 20x | 97.07% | 92.61% | 94.79% | 98.92% | 98.84% | 98.88% | 78.76% | 51.46% | 62.25% | 78.48% | 53.16% | 63.38% | 79.04% | 49.88% | 61.16% |  |  |  |
| Clair3 | HG004 | 20x | 97.07% | 92.56% | 94.76% | 99.11% | 98.92% | 99.02% | 77.39% | 51.07% | 61.53% | 75.49% | 52.71% | 62.08% | 79.35% | 49.56% | 61.01% |  |  |  |
| Clair3 | HG002 | 60x | 98.51% | 94.58% | 96.50% | 99.78% | 99.74% | 99.76% | 87.41% | 61.86% | 72.45% | 88.53% | 61.86% | 72.83% | 86.39% | 61.86% | 72.10% | 32460 | 35 | 680 |
| Clair3 | HG003 | 30x | 97.82% | 93.92% | 95.83% | 99.45% | 99.51% | 99.48% | 82.57% | 57.00% | 67.44% | 82.08% | 58.75% | 68.48% | 83.07% | 55.36% | 66.44% |  |  |  |
| Clair3 | HG004 | 30x | 98.02% | 93.80% | 95.86% | 99.66% | 99.58% | 99.62% | 82.69% | 56.14% | 66.88% | 80.50% | 58.18% | 67.55% | 84.97% | 54.26% | 66.23% |  |  |  |
| Clair3 | HG002 | 60x | 98.51% | 94.58% | 96.50% | 99.78% | 99.74% | 99.76% | 87.41% | 61.86% | 72.45% | 88.53% | 61.86% | 72.83% | 86.39% | 61.86% | 72.10% | 31502 | 35 | 575 |
| Clair3 | HG003 | 40x | 98.43% | 94.41% | 96.38% | 99.62% | 99.68% | 99.65% | 87.21% | 59.65% | 70.84% | 87.49% | 61.69% | 72.36% | 86.94% | 57.74% | 69.39% |  |  |  |
| Clair3 | HG004 | 40x | 98.55% | 94.27% | 96.37% | 99.76% | 99.69% | 99.72% | 87.27% | 58.95% | 70.37% | 85.18% | 60.95% | 71.06% | 89.43% | 57.11% | 69.70% |  |  |  |
| Clair3 | HG002 | 60x | 98.51% | 94.58% | 96.50% | 99.78% | 99.74% | 99.76% | 87.41% | 61.86% | 72.45% | 88.53% | 61.86% | 72.83% | 86.39% | 61.86% | 72.10% | 31465 | 35 | 501 |
| Clair3 | HG003 | 50x | 98.60% | 94.76% | 96.64% | 99.67% | 99.70% | 99.68% | 88.81% | 62.19% | 73.15% | 89.03% | 64.38% | 74.73% | 88.59% | 60.14% | 71.64% |  |  |  |
| Clair3 | HG004 | 50x | 98.74% | 94.70% | 96.68% | 99.80% | 99.74% | 99.77% | 89.04% | 61.81% | 72.97% | 87.23% | 63.88% | 73.75% | 90.90% | 59.92% | 72.22% |  |  |  |
| Clair3 | HG002 | 60x | 98.51% | 94.58% | 96.50% | 99.78% | 99.74% | 99.76% | 87.41% | 61.86% | 72.45% | 88.53% | 61.86% | 72.83% | 86.39% | 61.86% | 72.10% | 30725 | 35 | 458 |
| Clair3 | HG003 | 60x | 98.67% | 95.10% | 96.85% | 99.70% | 99.73% | 99.71% | 89.51% | 64.57% | 75.02% | 90.06% | 66.06% | 76.21% | 88.96% | 63.18% | 73.89% |  |  |  |
| Clair3 | HG004 | 60x | 98.88% | 95.08% | 96.94% | 99.81% | 99.76% | 99.79% | 90.60% | 64.56% | 75.39% | 89.38% | 66.95% | 76.56% | 91.84% | 62.36% | 74.28% |  |  |  |
| PEPPER | HG002 | 60x | 98.61% | 93.48% | 95.98% | 99.68% | 99.70% | 99.69% | 87.81% | 54.09% | 66.94% | 86.99% | 56.58% | 68.56% | 88.67% | 51.78% | 65.38% | 39971 | 35 | 3556 |
| PEPPER | HG003 | 10x | 54.01% | 83.86% | 65.70% | 54.89% | 91.51% | 68.62% | 41.90% | 33.37% | 37.15% | 37.34% | 37.35% | 37.35% | 48.92% | 29.65% | 36.92% |  |  |  |
| PEPPER | HG004 | 10x | 50.55% | 83.40% | 62.95% | 51.21% | 91.22% | 65.60% | 40.95% | 32.50% | 36.24% | 35.56% | 36.33% | 35.94% | 49.50% | 28.97% | 36.55% |  |  |  |
| PEPPER | HG002 | 60x | 98.61% | 93.48% | 95.98% | 99.68% | 99.70% | 99.69% | 87.81% | 54.09% | 66.94% | 86.99% | 56.58% | 68.56% | 88.67% | 51.78% | 65.38% | 25539 | 35 | 867 |
| PEPPER | HG003 | 20x | 87.96% | 92.13% | 90.00% | 89.30% | 98.97% | 93.89% | 73.03% | 46.95% | 57.15% | 71.07% | 51.42% | 59.67% | 75.35% | 42.77% | 54.56% |  |  |  |
| PEPPER | HG004 | 20x | 87.86% | 91.92% | 89.84% | 89.28% | 99.00% | 93.89% | 71.98% | 45.77% | 55.96% | 67.84% | 49.75% | 57.41% | 77.07% | 42.11% | 54.46% |  |  |  |
| PEPPER | HG002 | 60x | 98.61% | 93.48% | 95.98% | 99.68% | 99.70% | 99.69% | 87.81% | 54.09% | 66.94% | 86.99% | 56.58% | 68.56% | 88.67% | 51.78% | 65.38% | 23138 | 35 | 607 |
| PEPPER | HG003 | 30x | 96.97% | 93.24% | 95.07% | 98.52% | 99.54% | 99.03% | 81.08% | 51.65% | 63.10% | 78.89% | 55.90% | 65.44% | 83.63% | 47.68% | 60.73% |  |  |  |
| PEPPER | HG004 | 30x | 97.14% | 93.07% | 95.06% | 98.74% | 99.60% | 99.17% | 80.69% | 50.50% | 62.12% | 76.23% | 54.35% | 63.45% | 86.04% | 46.96% | 60.76% |  |  |  |
| PEPPER | HG002 | 60x | 98.61% | 93.48% | 95.98% | 99.68% | 99.70% | 99.69% | 87.81% | 54.09% | 66.94% | 86.99% | 56.58% | 68.56% | 88.67% | 51.78% | 65.38% | 21688 | 35 | 499 |
| PEPPER | HG003 | 40x | 98.05% | 93.64% | 95.79% | 99.38% | 99.66% | 99.52% | 84.53% | 53.90% | 65.83% | 82.31% | 58.18% | 68.17% | 87.08% | 49.90% | 63.45% |  |  |  |
| PEPPER | HG004 | 40x | 98.16% | 93.47% | 95.76% | 99.56% | 99.70% | 99.63% | 84.02% | 52.90% | 64.92% | 80.30% | 56.80% | 66.53% | 88.37% | 49.31% | 63.30% |  |  |  |
| PEPPER | HG002 | 60x | 98.61% | 93.48% | 95.98% | 99.68% | 99.70% | 99.69% | 87.81% | 54.09% | 66.94% | 86.99% | 56.58% | 68.56% | 88.67% | 51.78% | 65.38% | 21021 | 35 | 474 |
| PEPPER | HG003 | 50x | 98.41% | 93.86% | 96.08% | 99.57% | 99.69% | 99.63% | 86.66% | 55.40% | 67.59% | 84.83% | 59.53% | 69.96% | 88.71% | 51.54% | 65.20% |  |  |  |
| PEPPER | HG004 | 50x | 98.50% | 93.70% | 96.04% | 99.68% | 99.73% | 99.70% | 86.54% | 54.39% | 66.80% | 82.91% | 58.30% | 68.46% | 90.74% | 50.79% | 65.13% |  |  |  |
| PEPPER | HG002 | 60x | 98.61% | 93.48% | 95.98% | 99.68% | 99.70% | 99.69% | 87.81% | 54.09% | 66.94% | 86.99% | 56.58% | 68.56% | 88.67% | 51.78% | 65.38% | 20559 | 35 | 455 |
| PEPPER | HG003 | 60x | 98.64% | 94.02% | 96.27% | 99.61% | 99.72% | 99.67% | 88.70% | 56.35% | 68.91% | 86.80% | 60.31% | 71.17% | 90.82% | 52.65% | 66.66% |  |  |  |
| PEPPER | HG004 | 60x | 98.74% | 93.89% | 96.26% | 99.76% | 99.77% | 99.77% | 88.37% | 55.61% | 68.27% | 85.10% | 59.24% | 69.86% | 92.07% | 52.28% | 66.69% |  |  |  |

## Supplementary Table 6. HG002 trio benchmarking results while child’s coverage have lower coverage.

| Tool | Sample | Depth | Overall | | | SNP | | | Indel | | | Insertion | | | Deletion | | | # of MCV | *de novo* TP | *de novo* FP |
| --- | --- | --- | --- | --- | --- | --- | --- | --- | --- | --- | --- | --- | --- | --- | --- | --- | --- | --- | --- | --- |
|  |  |  | Precision | Recall | F1-Score | Precision | Recall | F1-Score | Precision | Recall | F1-Score | Precision | Recall | F1-Score | Precision | Recall | F1-Score |  |  |  |
| Clair3-Trio | HG002 | 10x | 97.18% | 90.44% | 93.69% | 98.44% | 96.88% | 97.66% | 84.04% | 49.61% | 62.39% | 82.87% | 50.00% | 62.37% | 85.16% | 49.25% | 62.41% | 6653 | 22 | 83 |
| Clair3-Trio | HG003 | 30x | 98.52% | 94.02% | 96.22% | 99.62% | 99.56% | 99.59% | 87.76% | 57.45% | 69.44% | 87.55% | 58.65% | 70.24% | 87.96% | 56.32% | 68.67% |  |  |  |
| Clair3-Trio | HG004 | 30x | 98.69% | 93.92% | 96.24% | 99.71% | 99.66% | 99.68% | 88.53% | 56.51% | 68.99% | 88.67% | 57.78% | 69.97% | 88.41% | 55.35% | 68.07% |  |  |  |
| Clair3 | HG002 | 10x | 82.24% | 83.33% | 82.78% | 83.89% | 91.08% | 87.33% | 62.00% | 34.25% | 44.12% | 66.85% | 34.89% | 45.85% | 57.95% | 33.65% | 42.58% | 47611 | 33 | 11218 |
| Clair3 | HG003 | 30x | 97.82% | 93.92% | 95.83% | 99.45% | 99.51% | 99.48% | 82.57% | 57.00% | 67.44% | 82.08% | 58.75% | 68.48% | 83.07% | 55.36% | 66.44% |  |  |  |
| Clair3 | HG004 | 30x | 98.02% | 93.80% | 95.86% | 99.66% | 99.58% | 99.62% | 82.69% | 56.14% | 66.88% | 80.50% | 58.18% | 67.55% | 84.97% | 54.26% | 66.23% |  |  |  |
| PEPPER | HG002 | 10x | 41.37% | 81.04% | 54.77% | 41.61% | 89.10% | 56.73% | 37.28% | 29.92% | 33.20% | 32.93% | 32.90% | 32.91% | 43.72% | 27.17% | 33.51% | 125058 | 31 | 87333 |
| PEPPER | HG003 | 30x | 96.97% | 93.24% | 95.07% | 98.52% | 99.54% | 99.03% | 81.08% | 51.65% | 63.10% | 78.89% | 55.90% | 65.44% | 83.63% | 47.68% | 60.73% |  |  |  |
| PEPPER | HG004 | 30x | 97.14% | 93.07% | 95.06% | 98.74% | 99.60% | 99.17% | 80.69% | 50.50% | 62.12% | 76.23% | 54.35% | 63.45% | 86.04% | 46.96% | 60.76% |  |  |  |

## Supplementary Table 7. Runtime and memory usage of different tools.

All results were tested on a machine with two 12-core Intel Xeon Silver 4116 processors using the 30x’s HG002 chr20 data.

| Tool | Average runtime for each sample (minutes) | Total runtime for trio (minutes) | Peak memory usage (gigabytes) |
| --- | --- | --- | --- |
| Clair3-Trio | - | 145m | 4.9G |
| Clair3 (v0.1-r6) | 42m | 126m | 2.5G |
| PEPPER (r0.4) | 48m | 144m | 27G |

ss

# Supplementary Notes

## Summary of methods tested that showed no or negligible improvement

(1) Adding position channels for trios: We added position channels in the Trio-to-Trio model to indicate the sample role in trios, including the child, parent1 and parent2 position channels. However, we observed that adding the position channel slightly decreased (-0.1%) the performance at the training terminal, when trained at chromosome 1 and tested at chromosome 20. We think that Clair3-Trio input, which is position order specific, is already encoded with position information, so it doesn’t need the position channels for trio variant calling.

(2) Training with all data, including the Mendelian inheritance violation (MCV) variants for Clair3-Trio models: We found marginal MCV variants in the GIAB true set (1442/4924197). We included the MCV sites in the training data and found no improvement in overall variant prediction accuracy, but higher predicted MCV (+382, tested at chromosome 20). Although including MCV variants enriched the training data, we found that including them may deter model training performance, as it results in a conflict with the MCVLoss function introduced in Clair3-Trio.

(3) Trio phasing: We phased alignment from trio based on the WhatsHap phasing with the pedigree option. We found that WhatsHap phasing with the pedigree option in ONT data results in a similar number of phased sites among different depths compared to individual phasing. Furthermore, extensive testing for trio phasing for calling variants in Clair3-Trio resulted in no significant difference compared to phasing alone. We believe that the individuals’ phasing module for ONT data is robust enough at present to gather information for variant calling.

## Data sources

### Reference genomes

#### GRCh38_no_alt

https://[ftp.ncbi.nlm.nih.gov/genomes/all/GCA/000/001/405/GCA_000001405.15_GRCh38/seqs_for_alignment_pipelines.ucsc_ids/GCA_000001405.15_GRCh38_no_alt_analysis_set.fna.gz](http://ftp.ncbi.nlm.nih.gov/genomes/all/GCA/000/001/405/GCA_000001405.15_GRCh38/seqs_for_alignment_pipelines.ucsc_ids/GCA_000001405.15_GRCh38_no_alt_analysis_set.fna.gz)

### GIAB Truth Variants

#### HG002 (NA24385), GRCh38, v4.2.1

https[://ftp-trace.ncbi.nlm.nih.gov/giab/ftp/release/AshkenazimTrio/HG002_NA24385_son/NISTv4.2.1/GRCh38/](ftp://ftp-trace.ncbi.nlm.nih.gov/giab/ftp/release/AshkenazimTrio/HG002_NA24385_son/NISTv4.2.1/GRCh38/)

#### HG003 (NA24149), GRCh38, v4.2.1

https[://ftp-trace.ncbi.nlm.nih.gov/giab/ftp/release/AshkenazimTrio/HG003_NA24149_father/NISTv4.2.1/GRCh38/](ftp://ftp-trace.ncbi.nlm.nih.gov/giab/ftp/release/AshkenazimTrio/HG003_NA24149_father/NISTv4.2.1/GRCh38/)

#### HG004 (NA24143), GRCh38, v4.2.1

https[://ftp-trace.ncbi.nlm.nih.gov/giab/ftp/release/AshkenazimTrio/HG004_NA24143_mother/NISTv4.2.1/GRCh38/](ftp://ftp-trace.ncbi.nlm.nih.gov/giab/ftp/release/AshkenazimTrio/HG004_NA24143_mother/NISTv4.2.1/GRCh38/)

### Oxford Nanopore (ONT) Sequencing Data

#### HG002 HD (NA24385), GRCh38_no_alt, 432.38-fold

https://s3-us-west-2.amazonaws.com/human-pangenomics/index.html?prefix=NHGRI_UCSC_panel/HG002/nanopore/Guppy_4.2.2/

#### HG003 Guppy 4.2.2 (NA24149), GRCh38_no_alt, 84.97-fold

https://s3-us-west-2.amazonaws.com/human-pangenomics/index.html?prefix=NHGRI_UCSC_panel/HG003/nanopore/Guppy_4.2.2/

#### HG004 GUPPY4.2.2 (NA24143), GRCh38_no_alt, 87.51-fold

[https://s3-us-west-2.amazonaws.com/human-pangenomics/index.html?prefix=NHGRI_UCSC_panel/HG004/nanopore/Guppy_4.2.2/](https://s3-us-west-2.amazonaws.com/human-pangenomics/index.html?prefix=NHGRI_UCSC_panel/HG004/nanopore/Guppy_4.2.2/GM24143_1-3_Guppy_4.2.2_prom.fastq.gz)

## Commands

### Read alignment using Minimap2 (v2.17-r941)

# Align ONT reads to GRCh38_no_alt

minimap2 -t ${THREADS} -aL -z 600,200 -x map-ont ref.fa input.fastq.gz | samtools view -bh -o output.unsorted.bam -

samtools sort -@ ${THREADS} -o output.sorted.bam output.unsorted.bam

samtools index -@ ${THREADS} output.sorted.bam

### BAM subsampling using Samtools (v1.10)

# Using ${FRAC} for both random seed and subsampling fraction

samtools view -@ ${THREADS} -s ${FRAC}.${FRAC} -b -o subsampled.bam ${BAM}

samtools index -@ ${THREADS} subsampled.bam

### Coverage calculation using Mosdepth (v0.2.9)

mosdepth -t ${THREADS} -n -x --quantize 0:15:150: output ${BAM}

### Clair3-Trio model training

Training section at: [https://github.com/HKU-BAL/Clair3-Trio](https://github.com/HKU-BAL/XXX).

### Running Clair3-Trio (v0.1)

${CLAIR3_TRIO_DIR}/run_clair3_trio.sh \

--bam_fn_c=${BAM_C} \

--bam_fn_p1=${BAM_P1} \

--bam_fn_p2=${BAM_P2} \

--ref_fn=${REF} \

--threads=${THREADS} \

--model_path_clair3="${MODEL_DIR_C3}" \

--model_path_clair3_trio="${MODEL_DIR_C3T}" \

--trio_model_prefix="${TRIO_M_PREFIX}" \

--output=${OUTPUT_DIR} \

--sample_name_c=${SAMPLE_C} \

--sample_name_p1=${SAMPLE_P1} \

--sample_name_p2=${SAMPLE_P2} \

### Running Clair3 (v0.1-r6)

docker run -it \

-v ${INPUT_DIR}:${INPUT_DIR} \

-v ${OUTPUT_DIR}:${OUTPUT_DIR} \

hkubal/clair3: v0.1-r6\

/opt/bin/run_clair3.sh \

--bam_fn=${INPUT_DIR}/input.bam \

--ref_fn=${INPUT_DIR}/ref.fa \

--threads=${THREADS} \

--platform="${PLATFORM}" \

--model_path="/opt/models/${PLATFORM}" \

--output=${OUTPUT_DIR}

### Running PEPPER (r0.4)

docker run --ipc=host \

-v "${INPUT_DIR}":"${INPUT_DIR}" \

-v "${OUTPUT_DIR}":"${OUTPUT_DIR}" \

kishwars/pepper_deepvariant:r0.4 \

run_pepper_margin_deepvariant call_variant \

-b "${BAM}" \

-f "${REF}" \

-o "${OUTPUT_DIR}" \

-p "${SAMPLE}" \

-t ${THREADS} \

--ont

### Benchmarking using hap.py (v0.3.12)

hap.py ${GIAB_BASELINE_VCF} output.vcf.gz \

-o ${OUTPUT_DIR}/happy \

-r ${REF} \

-f ${GIAB_CONFIDENT_BED} \

--threads ${THREADS} \

--pass-only \

--engine=vcfeval

### Merge VCF with BCFtools (v1.12)

M_VCF = merged_TRIO.vcf.gz

M_VCF_annotated = merged_annotated_TRIO_ann.vcf.gz

${BCFTOOLS} merge child.vcf.gz \

Parent1.vcf.gz \

Parent2. vcf.gz \

--threads 32 -f PASS -0 -m all| ${BCFTOOLS} view -O z -o ${M_VCF}

${BCFTOOLS} index ${M_VCF}

### Benchmarking using RTG tools (v3.12.1)

cat $PED

#fam-id ind-id pat-id mat-id sex phen

1 HG002 HG003 HG004 1 0

1 HG003 0 0 1 0

1 HG004 0 0 2 0

${RTG} mendelian -i ${M_VCF} -o ${M_VCF_annotated} --pedigree ${PED} -t ${REF_SDF_FILE_PATH}

### Computing the TP and FP of number of *de novo* variants

${BCFTOOLS} merge --threads 8 -f PASS -0 -m all ${GIAB Truth Variants HG002} ${GIAB Truth Variants HG003} ${GIAB Truth Variants HG004} | ${BCFTOOLS} view -O z -o ${TRIO_GIAB_MERGED }

${BCFTOOLS} index ${TRIO_GIAB_MERGED}

${CLAIR3_TRIO} Check_de_novo --call_vcf ${M_VCF} --ctgName chr20 --bed_fn PED --true_vcf $TRIO_GIAB_MERGED
